# Metabolomic Insights into Cross-Feeding Interactions Between *Priestia megaterium* PM and *Pseudomonas fluorescens* NO4: Unveiling Microbial Communication in Plant Growth-Promoting Rhizobacteria

**DOI:** 10.1007/s00248-025-02577-2

**Published:** 2025-07-17

**Authors:** Nompumelelo R. Sibanyoni, Lizelle A. Piater, Pavel Kerchev, Ntakadzeni E. Madala, Msizi I. Mhlongo

**Affiliations:** 1 Imbewu Metabolomics Research Group, Department of Biochemistry, Faculty of Science, University of Johannesburg, Auckland Park, 2006 South Africa; 2 Research Centre for Plant Metabolomics, Faculty of Science, University of Johannesburg, Auckland Park, 2006 South Africa; 3 Ubuntu Lab, Department of Biochemistry, Faculty of Science, University of Johannesburg, Auckland Park, 2006 South Africa; 4 Department of Stress, Development and Signalling in Plants, Estación Experimental del Zaidín, CSIC, Profesor Albareda 1, 18008 Granada, Spain; 5 Department of Biochemistry and Microbiology, Faculty of Science, Engineering and Agriculture, University of Venda, Thohoyandou, South Africa

**Keywords:** Cross-feeding, Chemical communication, Microbial interactions, Metabolomics, PGPR

## Abstract

Plant growth-promoting rhizobacteria (PGPR) engage in complex chemical exchange and signalling processes to enhance their survival, rhizosphere colonisation, and plant-beneficial roles. These microbial interactions are mediated by various chemical cues, including quorum sensing (QS) molecules, cyclic peptides, lipopeptides, nutrients, volatile organic compounds (VOC), and phytohormones. Cross-feeding, where one microorganism consumes metabolites produced by another, exemplifies direct chemical communication that shapes community dynamics and metabolic cooperation. However, the effects of cross-feeding among different PGPR strains remain insufficiently characterised. In this study, an LC–MS-based metabolomics approach, combined with multivariate statistical analysis, was employed to investigate metabolic perturbations induced by cross-feeding among PGPR strains. Growth curve analysis revealed that cross-fed PGPR exhibited growth patterns comparable to controls, with a slight reduction in biomass. Metabolic profiling indicated time-dependent shifts in the metabolic state of the cross-fed organisms, suggesting adaptive metabolic reprogramming in response to the donor-conditioned media. Multivariate analysis identified distinct metabolite alterations between cross-fed and control groups across different time points, highlighting the influence of nutrient availability on microbial growth dynamics. Notably, cross-fed groups showed decreased levels of primary metabolites such as amino acids and sugars alongside increased production of secondary metabolites, including surfactins, salicylic acid, and carboxylic acids. These secondary metabolites are implicated in plant growth promotion and defence, indicating their potential as natural biostimulants. The findings advance the understanding of PGPR interactions and chemical communication in the rhizosphere, supporting the development of sustainable agricultural practices by leveraging beneficial microbial interactions. Future research should explore these interactions within more complex microbial communities.

**Supplementary Information:**

The online version contains supplementary material available at 10.1007/s00248-025-02577-2.

## Background

The plant root microbiome, comprising a diverse community of microorganisms in and around plant roots, engages in complex interactions with the host plant [1]. This community includes both beneficial organisms such as plant growth-promoting rhizobacteria (PGPR), mycorrhizal fungi, and actinomycetes and harmful pathogens that can adversely affect plant growth. Beneficial microorganisms support plant health by enhancing nutrient uptake, improving stress tolerance, and suppressing plant diseases [2–4]. These interactions often involve symbiotic relationships mediated by root-secreted compounds, including sugars, proteins, and vitamins [5–7]. In contrast, pathogenic microorganisms can impair plant health by causing disease or inhibiting growth [1].

Among the beneficial microorganisms, PGPR have garnered significant attention for their ability to enhance plant growth, health, and productivity, while also conferring protection against biotic and abiotic stresses [5, 8–10]. These bacteria interact not only with plants but also with other PGPR strains, with most interactions reported to be mutualistic and cooperative in nature, reflecting beneficial microbial relationships [11–13]. Plants secrete root exudates to recruit and selectively enrich specific PGPR populations in the rhizosphere, thereby fostering associations that support growth, nutrient acquisition, and defence response [14, 15]. In return, PGPR provide a range of benefits to the host plant in exchange for root exudates, which act as both nutrient sources and signalling molecules [8, 11, 12].

Microbial interactions play a critical role in regulating various microbial functions, including adhesion, coordination of biofilm formation, inhibition of pathogenic microbes, motility within microbial communities, and the induction of resistance to biotic and abiotic stresses [16]. Effective communication is essential for microorganisms to interact with each other and the interactions occur via contact-dependent and contactless communication. Contact-dependent communication involves direct physical contact and serves as a regulatory mechanism by which bacteria coordinate the behaviour and development of neighbouring microbes [17]. In contrast, contact-independent communication relies on the production and release of various compounds, including antibiotics, nutritional metabolites, redox-active ions, peptides, hormones, root exudates, quorum sensing (QS) molecules, volatile organic compounds (VOCs), and other signalling molecules. These chemical cues are detected and interpreted by microbial communities to modulate collective behaviours and responses [3, 11, 12, 18–20].

Autoinducers initiate the chemical communication required for microbial interactions through a process known as quorum sensing (QS). QS facilitates microbial communication and plays a crucial role in regulating cross-feeding interactions [21]. It enables microorganisms to detect environmental changes and respond adaptively [18, 22]. Given its significant implications for agriculture, microbial chemical signalling has garnered substantial research interest. QS-mediated interactions regulate a wide range of processes, including biofilm formation, production of virulence factors, competence, conjugation, motility, sporulation, and antibiotic synthesis [6, 23]. However, microbial communities function within dynamic networks of interactions that underpin a complex web of interconnected metabolic processes [3, 24]. One of the key mechanisms driving these interactions is microbial cross-feeding (MCF), a fundamental process in microbial ecology. MCF refers to interactions between two or more microorganisms in which metabolic byproducts from one organism (the provider) serve as substrates for another (the beneficiary or receiver) [17, 25]. This occurs when compounds produced during the metabolism of one microorganism are further metabolised by another [24–26].

Microbial interactions are essential for colonisation, establishment, and persistence in diverse environments. These interactions typically involve environmental sensing, followed by the exchange of molecular and genetic information through a variety of molecules and mechanisms [3, 27]. Luzzatto-Knaan et al. [28] demonstrated that surfactin, produced by *Bacillus subtilis*, functions as an interspecies recruitment factor by promoting interactions with other soil microbes, such as *Paenibacillus dendritiformis*. In this context, surfactin serves both as a signalling molecule and a nutrient source. Moreover, the study showed that *P. dendritiformis* actively degrades *B. subtilis*-derived surfactins and accumulates the degradation products, which act as territorial markers. In a separate study, Andrić et al. [29] explored the interplay between *B. velezensis* and various *Pseudomonas* strains, revealing that *Bacillus*-derived surfactin can mitigate the toxicity of *Pseudomonas* cyclic lipopeptides. While these studies provide valuable insights into microbial chemical communication, the precise ecological functions of such interactions remain to be fully elucidated.

Metabolomics is a cutting-edge scientific approach that plays a crucial role in unravelling the intricate chemical communication within biological systems. This methodology enables comprehensive analysis and identification of the complete set of small molecules, or metabolites, present within an organism [15, 17]. It allows for deeper exploration of metabolic profiles in plants, microbes, and other systems, offering valuable insights into the chemical signals exchanged during their interactions [1, 15]. Despite advances in our understanding of microbial communication, the complex chemical signalling and exchange between microbial species in the rhizosphere particularly between PGPR strains remains poorly characterised. To address this knowledge gap, the present study employs metabolomics, which captures the unique chemical fingerprints of organisms, as a powerful tool to investigate chemical communication between two PGPR strains isolated from the same plant. By identifying and quantifying metabolites produced in specific environments, metabolomics offers critical insights into microbial cooperation and competition.

## Methods

### Microbial Species and Culture Conditions

Two bacterial strains were chosen based on their specific traits, community stability, and experimental design. The two selected PGPR strains, *P. megaterium* PM and *P. fluorescens* NO4 were chosen due to previous research demonstrating their ability to promote plant growth and enhance resistance to both biotic and abiotic stresses. The study by Rudolph [30] describes the pure cultures of the two bacterial isolates. *P. megaterium* PM (formerly *Bacillus megaterium* BM) was reclassified in 2020 based on comprehensive genomic and phylogenetic analyses that revealed its distinct evolutionary position within the *Bacillus* genus [31]. This strain has been shown to enhance root colonisation and promote the growth of tomato and wheat plants [32]. Mhlongo et al. [16] further demonstrated that *P. fluorescens* NO4 contributes to plant health by promoting growth and activating defence mechanisms through the production of secondary metabolites and aromatic amino acids. The two PGPR strains were obtained from glycerol stock samples stored at the University of Johannesburg Department of Biochemistry. Bacterial cultures were initially grown on LB agar (Merck, South Africa) plates, then cultured in liquid M9 minimal media for microbial growth. The medium composition per liter included 25.6 g sodium phosphate dibasic (Na₂HPO₄), 6.0 g potassium phosphate monobasic (KH₂PO₄), 1.0 g sodium chloride (NaCl), 2.0 g ammonium chloride (NH₄Cl), supplemented with 10.0 g glucose as the primary carbon source and 2.0 g malic acid to support additional metabolic activity. M9 minimal media was used to achieve a defined and controlled environment for investigating microbial interactions without interference from complex and undefined nutrients.

### Growth and Maintenance of Bacterial Culture

The PGPR strains *P. fluorescens* NO4 and *P. megaterium* PM were cultured in 30 mL liquid M9 media in 50 mL Erlenmeyer flasks. The cultures were grown from LB agar plates and incubated in a shaking incubator at 160 rpm and 30 °C until they reached an optical density of 1 at 600 nm. The bacterial cultures were then separated into donor media and receiver species groups to investigate cross-feeding interactions between the two strains. For the donor, actively growing bacterial cultures were centrifuged at 4700 rpm for 15 min. The supernatants were filter-sterilised (0.22 µm filter membranes) into 50 mL Erlenmeyer flasks in preparation for the receiver species. The receiver species cultures were diluted to an OD of 0.1 at 600 nm and were transferred into their respective donor supernatant for cross-feeding. All cultures were incubated at 30 °C, shaking at 160 rpm for 36 h.

### Design of the Experiment, Culture Harvest, and Metabolite Extraction for Microbial Cross-Feeding (MCF)

**Fig. 1:**
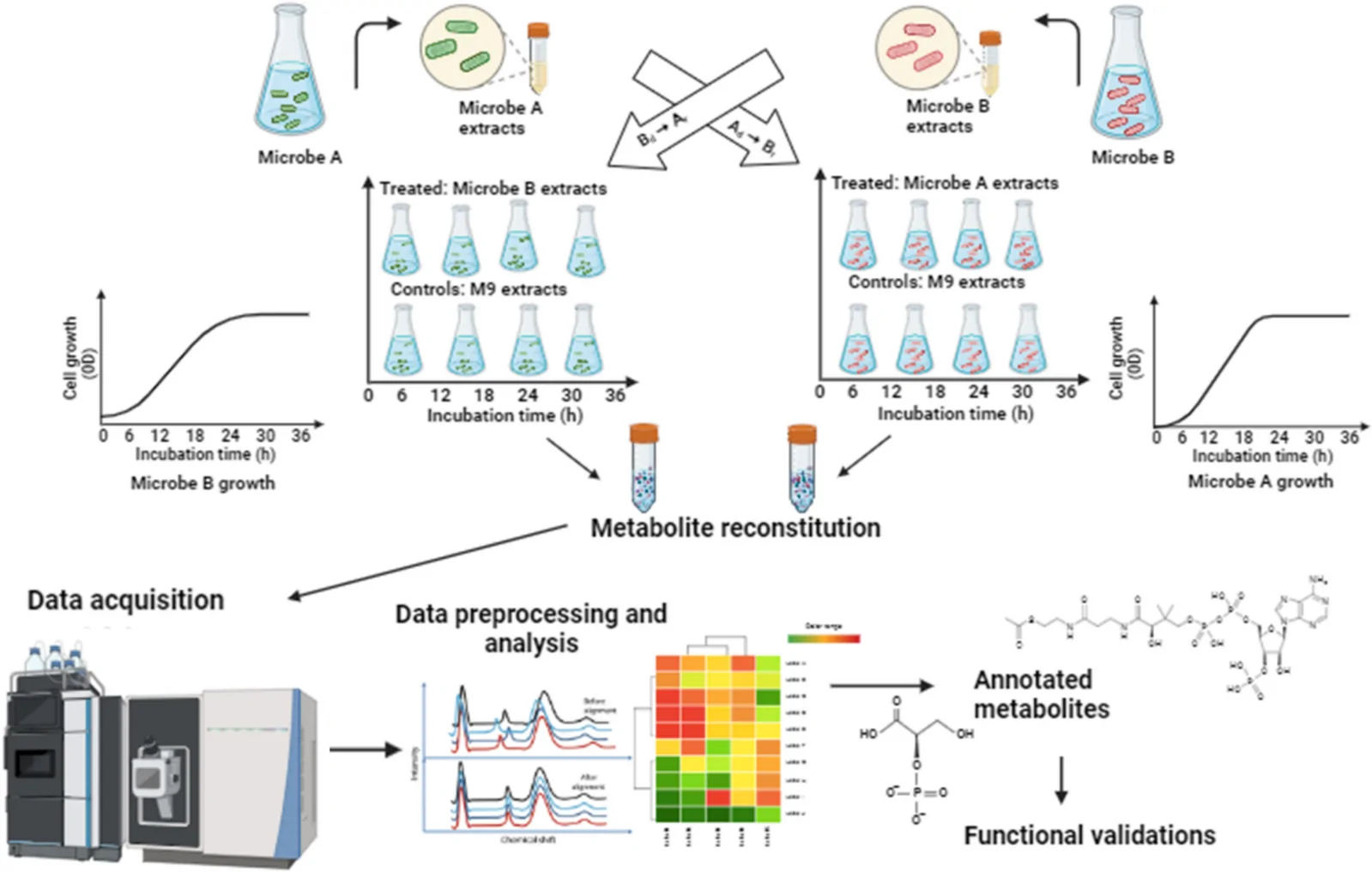
The experimental design illustration of the microbial cross-feeding (MCF) of *P. megaterium* PM and *P. fluorescens* NO4 PGPR species*.* Cross-feeding interactions were established between *P. megaterium* PM (microbe A, receiver) and *P. fluorescens* NO4 (microbe B, donor) in MCF-1, and vice versa in MCF-2, using liquid M9 medium pre-conditioned with donor culture supernatant. Growth was monitored at 6 h intervals (0, 6, 12, 18, 24, 36 h) via OD600 measurements. Cultures were centrifuged, and supernatants were stored at − 80 °C after freeze-drying. Samples were reconstituted, and data acquisition was followed on the ultra-performance liquid chromatography quadrupole time-of-flight mass spectrometry (UPLCQ-TOF MS) instrument. Data processing and analysis were performed, metabolites were annotated on MSDIAL and GNPS, and functions were validated

Three independent biological replicates for microbial cross-feeding (MCF) were conducted, along with appropriate controls. MCF interactions between *P. megaterium* PM and *P. fluorescens* NO4 were performed in liquid M9 medium conditioning before inoculation. The diluted culture of *P. megaterium* PM was extracted as a receiver and cross-fed to *P. fluorescens* NO4 as a donor in MCF-1 (NO4_PM) (Fig. 1). Conversely, the diluted culture of *P. fluorescens* NO4 as a receiver was extracted and cross-fed to *P. megaterium* PM as a donor in MCF-2 (PM_NO4). The control samples (single cultures of the strains) were added using the M9 medium (30 mL) to normalise the growth effect. The growth of the MCF-treated and control replicates was examined at 6 h intervals (0, 6, 12, 18, 24, and 36) for variation of viability (cell viability at OD600, Tables S1 and S2) and metabolite identification. Growth curves were drawn for all MCFs against their controls. The microbial cultures were centrifuged at 4700 rpm for 15 min. The supernatant was transferred into 15 ml Falcon tubes and stored in a - 80 °C freezer, and the samples were freeze-dried. The dried samples were reconstituted with 0.1% formic acid (FA) in 50% methanol, and then, the samples were further diluted in a 1:1 ratio with 0.1% FA in 50% methanol for LC–MS/MS analysis.

### Liquid Chromatography-Mass Spectrometry Data Acquisition

The extracts were analysed using the ACQUITY ultra-high performance liquid chromatography coupled with SYNAPT XS quadrupole time-of-flight Mass Spectrometer (Waters, Milford, MA, USA). Chromatographic separation was performed using an ACQUITY™ PREMIER HSS T3 1.8 μm column. The mobile phase consisted of solvent A (water with 0.1% formic acid (FA) and 2.5% isopropyl alcohol (IPA)) and solvent B (acetonitrile with 0.1% formic acid and 2.5% IPA). The gradient elution program was as follows: 100% A to 0.0% B for initial at 0 min; 100% A to 0.0% B for 0 to 1 min; 10% A to 90% B for 1 to 15 min; 1.0% A to 99.0% B for 15 to 15.10 min; 1.0% A to 99.0% B for 15.10 to 17 min; 100% A to 0.0% B for 17 to 17.10 min; followed by a 100% A to 0.0% B for 17.10 to 20 min. The injection volume was set to 2 µL, with a flow rate of 0.4 mL/min. The run time was 21 min. The spectrum acquisition was performed in both positive and negative ion modes using a data-independent acquisition (DIA) approach analogous to MS^E^, covering a mass range of 100–1500 Da. The method alternated collision energies from low (20 eV) to high (40 eV) to acquire comprehensive fragmentation data without precursor ion preselection. The following source and instrument parameters were applied: capillary sample injection was done at 0.6 kV, with a cone voltage at 30 V, source temperature at 120 °C, desolvation temperature were maintained at 120 °C and 450 °C, desolvation gas flow: 600 L/h, cone gas flow 50 L/h, and nebuliser gas flow 6.5 Bar. The scan time was 0.1 s per scan with zero interscan delay, enabling sufficient data points across chromatographic peaks. Data acquisition was performed in continuum mode at a resolution of approximately 10,000. The lock mass calibration was infused at 5 µL/min via the Lockspray interface, with a capillary voltage of 1.3 kV for the lock mass. The system was controlled by MassLynx software version 4.2 SCN1028.

### Data (Pre)-processing and Multivariate Statistical Analysis

The .raw vendor (Waters) MS/MS format files acquired in ESI positive were converted to analysis base file (ABF) file format on the Reifys Abf converter software5 that is compatible with the Mass Spectrometry-Data Independent Analysis (MS-DIAL) software. MSDIAL is a software tool that processes mass spectrometry data by deconvolution on both data-dependent acquisition (DDA) and.data-independent acquisition (DIA) datasets, handling both centroid and profile data formats [33]. This software integrates key sources of information, including accurate mass, retention time (Rt) prediction, isotope ratios, and MS/MS fragment matching to assist in compound identification and annotation [33]. The converted ABF data files were imported into the MS-DIAL software for processing. Data processing was carried out using the following specific parameters: mass accuracy 0.01 Da MS1 and 0.025 Da MS2 tolerance, with a retention time window from 0.7 to 17 min. The mass range was analysed from 100 to 1500 Da. For the peak detection, a minimum peak height of 2500 amplitude was applied, with a mass slice width of 0.1 Da. For data deconvolution, a sigma window value of 0.5 and a 0 MS/MS abundance cut-off were applied. Alignment parameters included a retention time (Rt) tolerance of 0.02 min with one of the QC samples used as a reference file for alignment.

The resultant area data file from MSDIAL was transposed into a matrix file and imported into MetaboAnalyst 6.0 (https://www.metaboanalyst.ca/MetaboAnalyst/ModuleView.xhtml) software online [34] The one-factor statistics generic format was used in tab-delimited (txt). Mean normalisation, log transformation, and Pareto scaling were applied for multivariate statistical analysis (MVDA). MVDA involves the simultaneous observation and analysis of multiple outcome variables to identify patterns, relationships, and dependencies among them. This approach provides deeper insights compared to univariate or bivariate analyses, which focus on one or two variables, respectively [35]. The common techniques used in MVDA include principal component analysis (PCA), partial least squares discriminant analysis (PLS-DA), and orthogonal-partial least square discriminant analysis (OPLS-DA). This study employed PLS-DA, a supervised method that reduces data complexity while combining partial least squares regression with discriminant analysis. It is particularly useful for classifying observations based on their feature profiles, making it ideal for metabolomics and other high-dimensional datasets [36].

### Molecular Networking and Metabolite Annotation

The processed files from MSDial were converted into GNPS-compatible formats (GnpsMgf and GnpsTable) and uploaded to the GNPS2 platform along with metadata files that detailed sample properties like treatment and harvesting time. The Global Natural Product Social Molecular Networking 2 (GNPS 2) website was used to create molecular networks using the online workflow (https://gnps2.org/workflows). The mass tolerance for the precursor ions and MS/MS fragment ion tolerance was set at 0.5 Da. A minimum of six matched peaks and a cosine similarity scores above 0.7 were required to generate the molecular network. Cytoscape was used for network visualisation, with a maximum molecular family size of 100 sets.

Metabolite features annotation was performed through a combination of automated and manual approaches. First, automated annotations were obtained through MS-DIAL and GNPS2. MSDIAL utilises MSP spectral libraries for metabolomics, incorporating publicly available MS/MS libraries to facilitate automated annotations. In GNPS2, the Feature-Based Molecular Network (FBMN) workflow was used to generate automated annotations. Using the following specific parameters: the precursor and fragment ion tolerance were both set at 0.05, the min cosine at 0.6, and the min matched peaks at 4 for networking parameters, then everything else was used in default mode. Secondly, manual annotation was conducted by searching for molecular formulas MF and *m/z* values of metabolites previously reported in the literature to be produced by the microbes. These values were cross-referenced with GNPS cluster summaries and MSDIAL. Finally, both automated and manual annotated metabolites were verified against various databases, including PubChem (https://pubchem.ncbi.nlm.nih.gov/, accessed on 4–10 September 2024), KEGG (https://www.genome.jp/kegg, accessed on 4–10 September 2024), and Massbank (https://massbank.eu/MassBank/, accessed on 4–10 September 2024) to confirm the molecular formula (MF), chemical name, *m/z*, and fragmentation patterns. Therefore, this annotation method met the Metabolomics Standards Initiative (MSI) levels 2 and 3 of the MSI [37]. No pure standards were used to identify and confirm metabolites.

### Annotation Confirmation and Interpretation Through Pathway Analysis

The Kyoto Encyclopedia of Genes and Genomes (KEGG) (https://www.genome.jp/kegg/, accessed on 4 September 2024) was used to confirm the annotated metabolites and map them to biochemical pathways, and Chemspider (http://www.chemspider.com/, accessed on 12 September 2024). The observed MVDA data was followed by the biological interpretation of the annotated metabolites and used to construct the metabolic pathway analysis (MetPA) in MetaboAnalyst 6.0, powered by KEGG, to visualise the impacts of microbial interactions on the metabolic reprogramming on the metabolome and growth of the receiver species.

## Results

### The Influence of Microbial Cross-Feeding on the Growth of the Receiver Species Through Analysis of Growth Curves

**Fig. 2:**
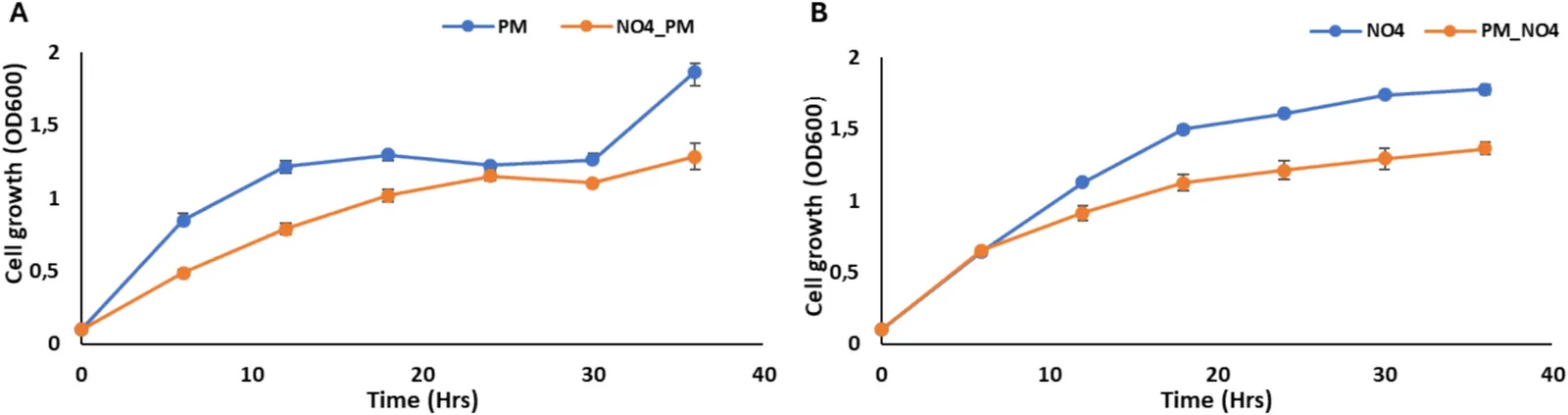
Microbial growth curves illustrating the effect of PGPR-PGPR cross-feeding on the growth of each respective receiver microbe. **A** *P. megaterium* PM as the receiver species, *P. fluorescens* NO4 as the donor species, **B** *P. fluorescens* as the receiver species, and *P. megaterium* PM as the donor species

Microbial growth curves provide a detailed graphical representation of growth patterns over time [38]. It provides detailed insights into microbial growth, offering insights into how microbes respond to varying experimental conditions over time. Microbial growth curves typically consist of four distinct phases: lag phase, exponential (log) phase, stationary phase, and death (decline) phase [39, 40]. The microbial growth curves observed in this study (Fig. 2) exhibited a consistent trend across all treatments (NO4_PM and PM_NO4), characterised by a slight decrease in the growth of all cross-fed microbes. Cross-feeding between the respective PGPR strains showed no significant impact, as their growth patterns closely resembled those of their respective controls, with only a minor reduction observed (Fig. 2A and B).

### Annotation of Metabolites Involved in the Microbial Cross-Feeding Chemical Communication

To investigate and profile the effects of cross-feeding on growth and metabolic perturbations over time, this study employed an untargeted metabolomics approach. Metabolite annotation was performed using a hybrid strategy that combined automated computational workflows with manual curation. Automated annotations were conducted within both the GNPS and MS-DIAL platforms. A comprehensive manual curation step was then applied to refine and confirm the metabolite identifications. The metabolites were validated against spectral databases (PubChem, MassBank, and KEGG) to ensure a high degree of confidence in the annotations. As a result, a total of 42 metabolites from diverse classes, spanning both primary and secondary metabolic pathways, were confidently identified through the combined automated and manual annotation process. The complete list of annotated metabolites is presented in Table S2.

**Fig. 3:**
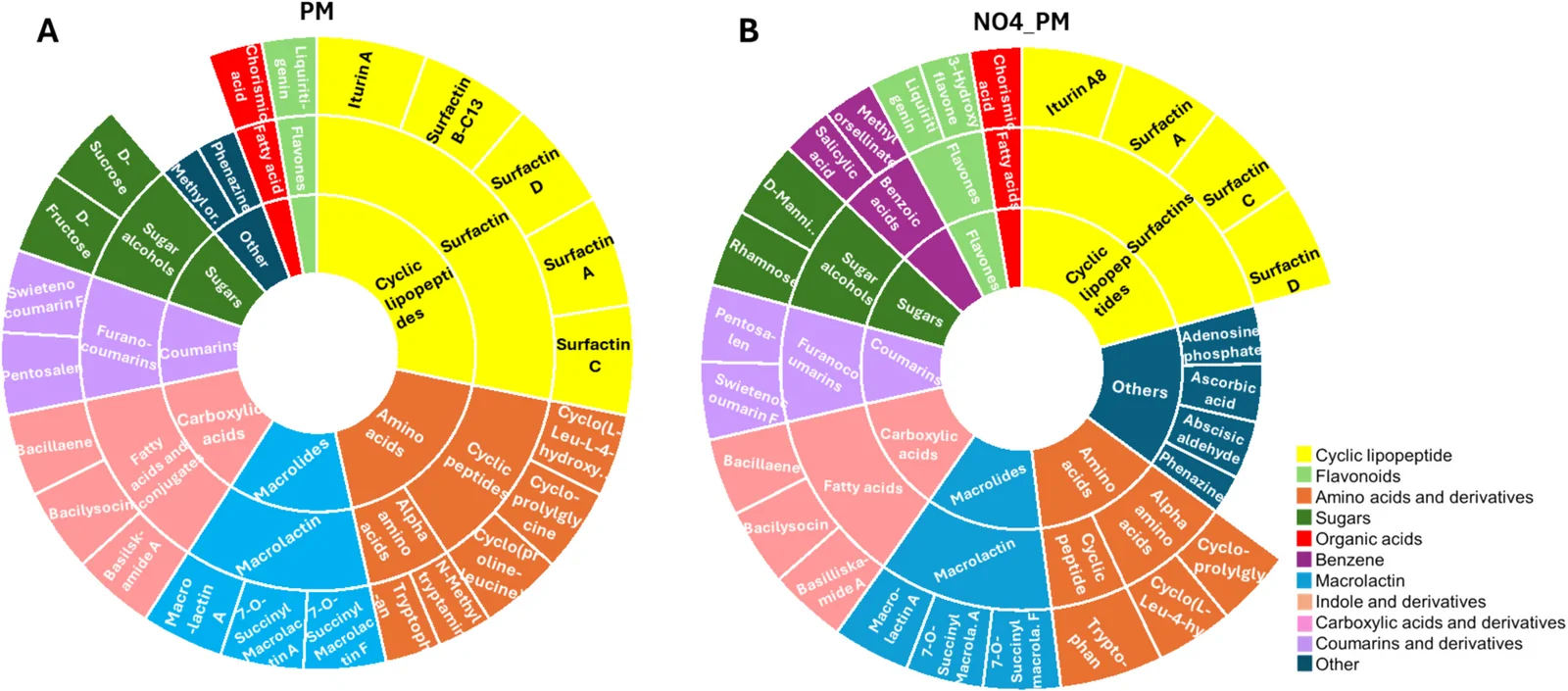
Sunburst plots representing the classification of all the annotated metabolites for the effect of *P. fluorescens* NO4 on *P. megaterium* PM. **A** *P. megaterium* PM acts as the control for the cross-fed samples. **B** *P. megaterium* PM as the receiver species, and *P. fluorescens* NO4 as the donor species. The annotated chemical classes include amino acids, cyclic lipopeptides, carboxylic acids and derivatives, benzene and substituted derivatives, coumarins and derivatives, flavonoids, macrolides, sugars, organic acids, indole, and others. Among these, amino acids (orange), cyclic lipopeptides (yellow), macrolides (light blue), and carboxylic acids (pink) were the dominant classes

**Fig. 4:**
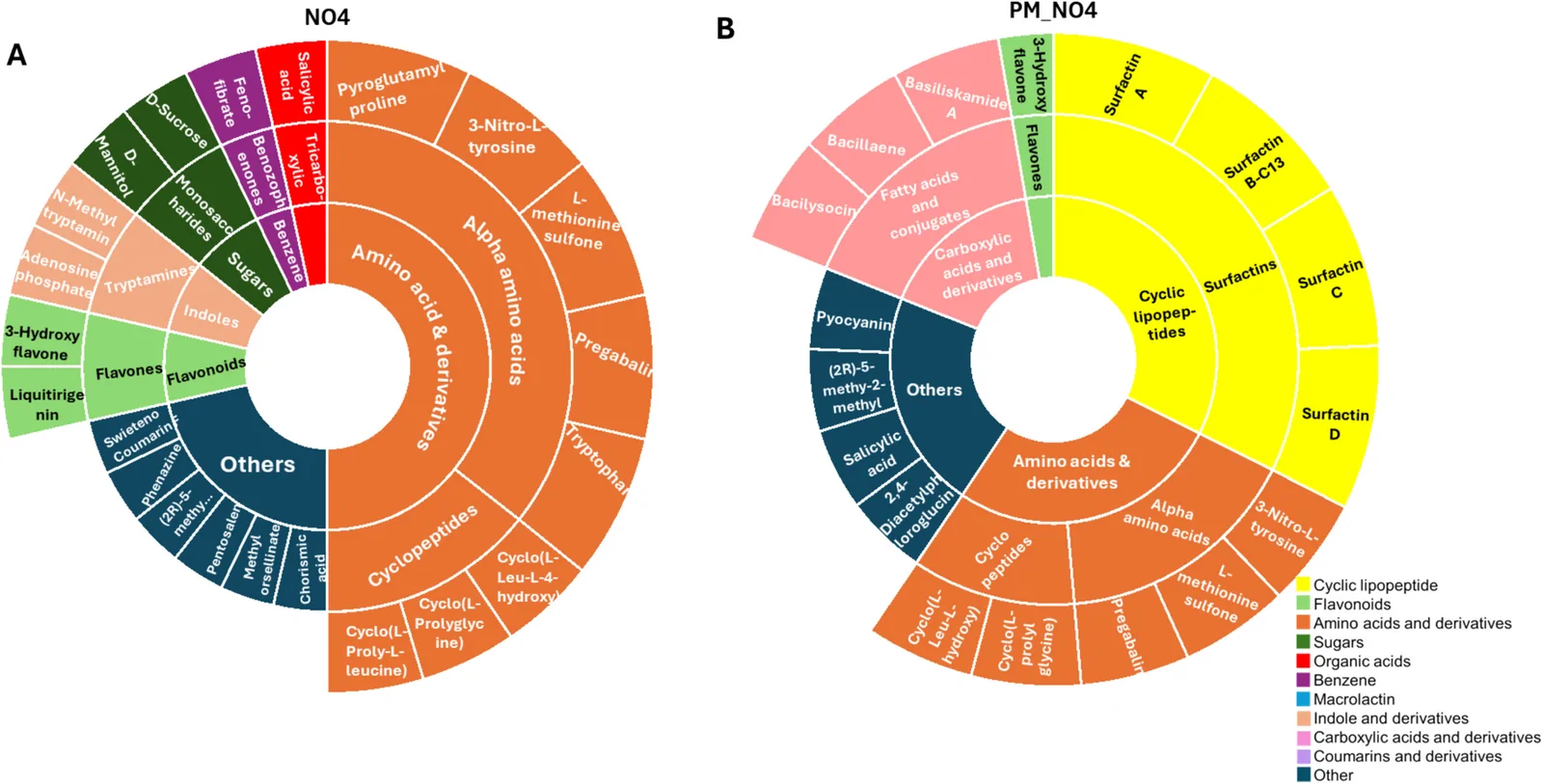
Sunburst plots representing the classification of all the annotated metabolites for the effect *P. megaterium* PM on *P. fluorescens* NO4. **A** *P. fluorescens* NO4 as the receiver species, and *P. megaterium* PM as the donor species. **B** *P. megaterium* PM as the receiver species, and *P. fluorescens* NO4 as the donor species. The annotated chemical classes include amino acids, cyclic lipopeptides, carboxylic acids and derivatives, benzene and substituted derivatives, coumarins and derivatives, flavonoids, macrolides, sugars, organic acids, indole, and others. Among these, amino acids (orange), cyclic lipopeptides (yellow), and carboxylic acids (pink) were the dominant classes

The annotated metabolites were visualised using sunburst plots (Figs. 3 and 4) to illustrate their distribution across treatments and the reciprocal effects of *P. fluorescens* NO4 and *P. megaterium* PM. Figure 3 compares the metabolite profile of *P. megaterium* PM under control conditions (Fig. 3A) and when exposed to the donor medium of *P. fluorescens* NO4 (NO4_PM, Fig. 3B). Cross-feeding (NO4_PM) resulted in a reduction of amino acids (e.g., N-methyltryptamine, cyclo-(proline-leucine)), sugars (fructose, sucrose), and surfactin (Surfactin B-C13). In contrast, flavonoids (e.g., 3-hydroxyflavone), benzene derivatives (e.g., salicylic acid), and other metabolites (adenosine phosphate, ascorbic acid, abscisic aldehyde) increased. Similarly, Fig. 4 shows *P. fluorescens* NO4 metabolite profiles under control (Fig. 4A) and cross-fed conditions using *P. megaterium* PM donor medium (PM_NO4, Fig. 4B). Cross-feeding led to a decrease in amino acids (pyroglutamyl-proline, tryptophan), sugars (sucrose, mannitol), and flavonoids (liquiritigenin), alongside the emergence of cyclic lipopeptides (Surfactins A, B-C13, C, D) and carboxylic acids (Basiliskamide A, Bacillaene, Bacilysocin). Indole compounds such as adenosine phosphate and N-methyltryptamine were absent in PM_NO4. These results highlight species-specific metabolic responses: *P. megaterium* PM is characterised by macrolactins, surfactins, coumarins, and carboxylic acids, while *P. fluorescens* NO4 is distinguished by indoles, benzene derivatives, and select amino acids. Overall, the findings underscore dynamic biochemical reprogramming during cross-feeding interactions.

The molecular networks (Fig. S1) provided an additional visual representation of the metabolite classes, facilitating an intuitive understanding of their distribution and their prevalence. The analysis revealed that the most predominant metabolite classes included amino acids, sugars, organic acids, cyclic lipopeptides, carboxylic acids, flavonoids, indoles, benzene derivatives, macrolides, and coumarins. Notably, cyclic lipopeptides and macrolactins were exclusively detected in interactions involving *P. megaterium* PM (Figs. 3A and B, 4B), suggesting that these metabolites are unique to this species. In contrast, indoles and benzene were exclusively observed in *P. fluorescens* NO4 (Fig. 4A) control samples, indicating that these compounds are produced by *Pseudomonas* species rather than by *Priestia* strains. Meanwhile, other metabolites were shared across all treatments, highlighting the complexity of microbial metabolic exchanges.

### Multivariate Data Analyses of Key Metabolites Influencing PGPR-to-PGPR Cross-Feeding Interactions

**Fig. 5:**
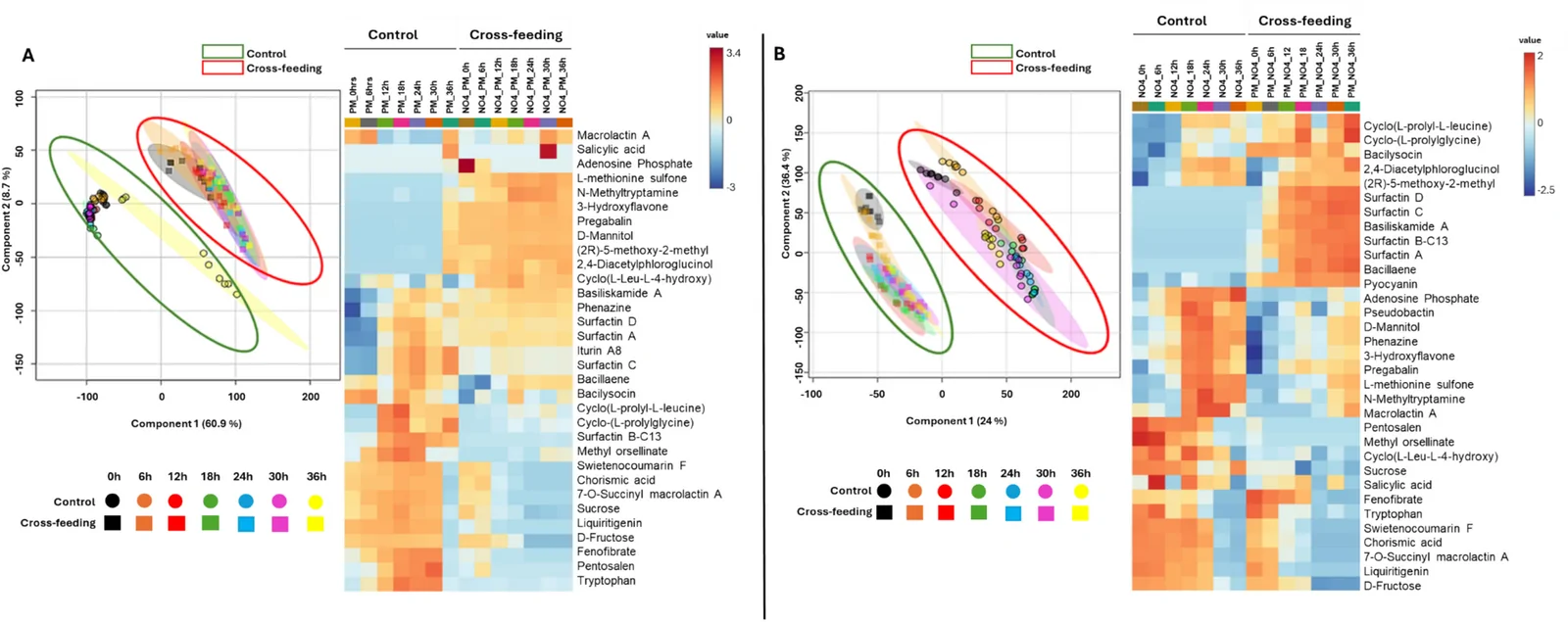
Time-correlated chemometric analysis of the supervised PLS-DA score plots for the metabolite profiles of the cross-feeding treatments and corresponding heatmap based on the PLS-DA module highlighting the significantly discriminant metabolites between PGPR-PGPR cross-cultures over 36 h. **A** *P. fluorescens* as the receiver species, and *P. megaterium* PM as the donor species. **B** *P. megaterium* PM as the receiver species, *P. fluorescens* NO4 as the donor species

Multivariate data analysis (MVDA), particularly partial least squares-discriminant analysis (PLS-DA), is a key chemometric approach for reducing data complexity and identifying significant patterns in metabolomics. PLS-DA is effective for binary and multi-class classification, identifying metabolites that differentiate between groups [9, 39], complementing the unsupervised insights from principal component analysis (PCA). This study primarily used PLS-DA to visualise overall differences across multiple sample groups, control and cross-fed over time. While PLS-DA can identify specific discriminant biomarkers even with multiple groups, in this study, they were focused on broader group separation. Heatmaps were also employed to visually represent metabolite abundance, with rows indicating metabolites and columns indicating sample conditions. The models, generated using MetaboAnalyst 6.0 (Fig. 5), highlight distinct metabolomic differences between cross-fed and control samples. Specifically, Fig. 5A shows *P. fluorescens* NO4 as the donor and *P. megaterium* PM as the receiver (NO4_PM). Cross-fed samples clustered distinctly from controls across all time points. A unique cluster observed in the 36-h control sample suggests metabolic shifts due to bacterial growth and secondary metabolite production.

The heatmap in Fig. 5A supports the PLS-DA results, highlighting distinct metabolic profiles between control and cross-fed samples. Several metabolites were uniquely present or absent between groups, indicating a clear metabolic shift. Cross-fed *P. megaterium* PM showed reduced levels of amino acids and sugars correlated with decreased growth (Fig. 4A), while Surfactin levels increased across all time points, suggesting a response to cross-feeding. Some metabolites were treatment-specific, while others appeared consistently, reflecting a complex and dynamic metabolic response. In Fig. 5B, where *P. megaterium* PM is the donor and *P. fluorescens* NO4 is the control, the control samples cluster tightly over time, distinctly separated from the cross-fed PM_NO4 samples, indicating sustained metabolomic perturbations. Despite the different species roles, this pattern mirrors Fig. 5A, reinforcing the time-dependent metabolic shifts driven by cross-feeding. These findings offer critical insights into microbial interaction dynamics, highlighting the biochemical basis of cooperation and competition [41].

Variable Importance in Projection (VIP) score plots generated via MetaboAnalyst identified key discriminatory metabolites (VIP > 0.5) distinguishing cross-fed samples from controls. Figure S2 illustrates metabolic shifts driven by PGPR interactions between *P. megaterium* PM and *P. fluorescens* NO4. In Fig. S2A, where NO4 is the donor and PM the receiver, metabolites such as (2R)−5-methoxy-2-methyl-2,3,8,9-tetrahydrofuro[2,3-h]chromen-4-one, pregabalin, and 2,4-diacetylphloroglucinol emerged in cross-fed samples, while liquiritigenin and D-fructose disappeared, indicating significant metabolic changes. In Fig. S2B, with PM as the donor and NO4 as the receiver, five metabolites (Surfactin A, D, C, B-C15, and Basiliskamide) were unique to cross-fed samples, suggesting a distinct metabolic response to donor-derived cues, likely aiding microbial adaptation in the rhizosphere.

### Metabolic Reprogramming of Respective Treatments at Different Growth Stages

**Fig. 6:**
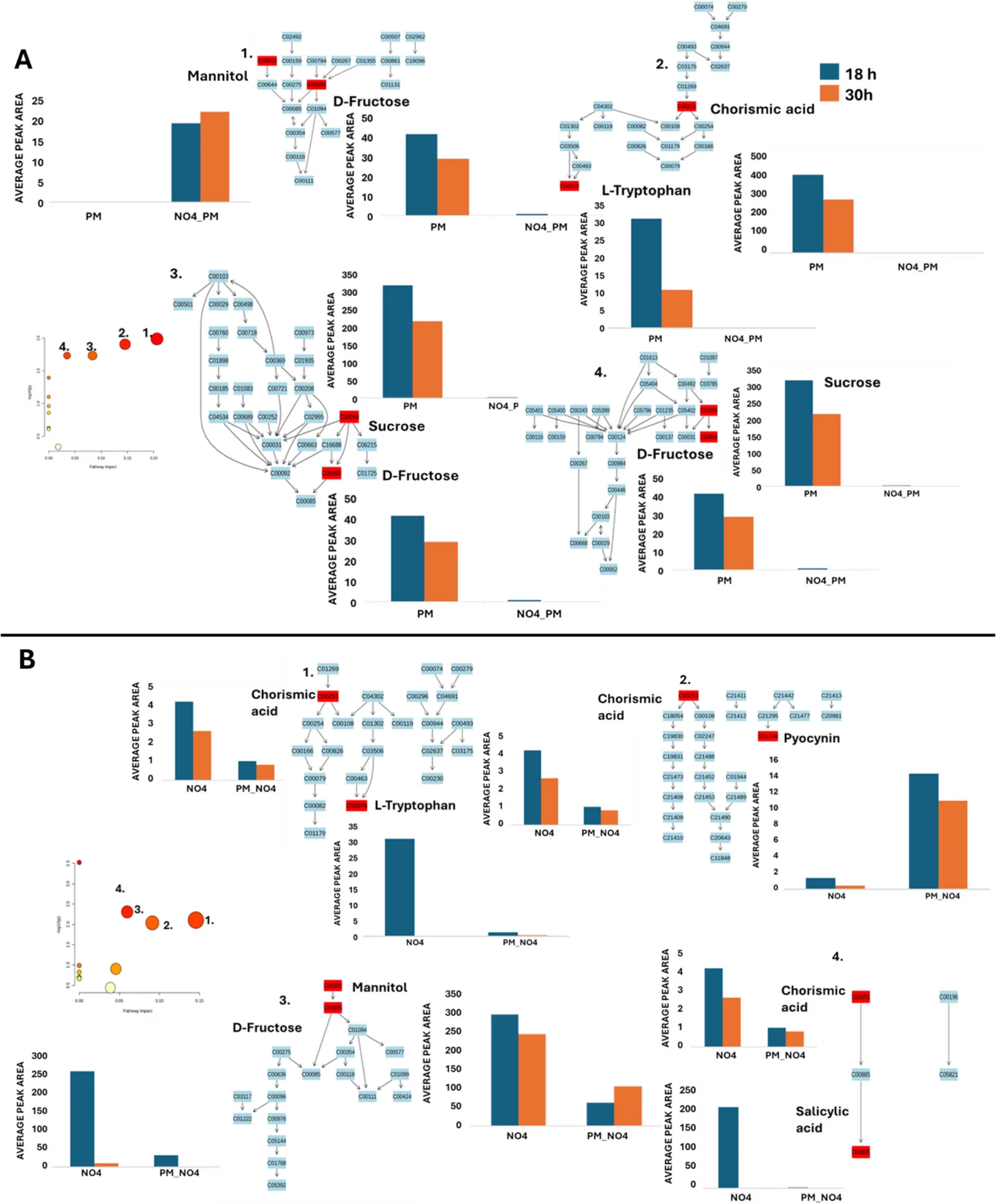
A summary of metabolic pathways analysis generated in MetPA illustrating significant and impactful pathways, along with relative quantification of altered metabolites of altered metabolites resulting from PGPR cross-feeding. The metabolome view presents mapped metabolic pathways, where the y-axis ranks pathways based on *p*-values (indicating statistical significance), while the x-axis ranks them by pathway impact values (reflecting biological relevance). Pathway impact values refer to the cumulative contribution of matched metabolite nodes, with a maximum importance score of 1. The accompanying bar graphs depict metabolic reprogramming at two distinct growth phases: exponential (18 h) and stationary (30 h). In **A**, *P. megaterium* PM serves as the donor species in **A**, while *P. fluorescens* NO4 is the donor species in **B**

The impact of microbial interactions on the metabolome and growth of the receiver species was further assessed using Metabolic Pathway Analysis (MetPA) via MetaboAnalyst 6.0, based on the annotated metabolites (Table S2). Pathway enrichment was performed using metabolite names, selecting KEGG prokaryotic pathways: *Priestia megaterium* QM B1551 [bmq] for *P. megaterium* PM and *Pseudomonas aeruginosa* PAO1 [pae] for *P. fluorescens* NO4. MetPA integrates statistical and pathway topology analyses to identify significantly altered and high-impact pathways. In this analysis, matched metabolites are shown in red, unmatched in blue, and pathways are ranked by *p*-value (-log10 scale, y-axis) and impact score (x-axis) (Fig. 6A and B).

Figure 6 summarises the pathway alterations resulting from PGPR interactions. In Fig. 6A, with *P. fluorescens* NO4 as the donor and *P. megaterium* PM as the receiver (NO4_PM), four major pathways were significantly affected: (1) fructose and mannose metabolism, (2) phenylalanine, tyrosine, and tryptophan biosynthesis, (3) galactose metabolism, and (4) starch and sucrose metabolism. Notably, the latter three pathways were enriched in control samples, with tryptophan, chorismic acid, sucrose, and fructose showing high abundance at 18 h but declining by 30 h. In contrast, mannitol, associated with fructose and mannose metabolism, accumulated exclusively in cross-fed samples over time, suggesting a metabolic adaptation to cross-feeding and a shift in pathway activity driven by microbial interaction.

In Fig. 6B, *P. megaterium* PM acts as the donor, and *P. fluorescens* NO4 as the receiver (NO4_PM). Four key pathways were significantly affected: (1) phenylalanine, tyrosine, and tryptophan biosynthesis, (2) phenazine biosynthesis, (3) fructose and mannose metabolism, and (4) siderophore group nonribosomal peptide biosynthesis. Similar to Fig. 6A, pathways 1, 3, and 4 were enriched in control samples, with high levels of chorismic acid, tryptophan, mannitol, fructose, and salicylic acid at 18 h, which declined by 30 h. Pathways 2 and 3 showed distinct trends, as pyocyanin accumulated more in cross-fed samples, while salicylic acid was higher in controls, though both declined over time.

These pathways underscore the role of sugar and amino acid metabolism in mediating PGPR interactions. Growth curve and heatmap data (Figs. 2A and B, 5A and B) further support a slight growth reduction in the cross-fed PM strain, suggesting that metabolite availability influences both cooperation and competition in microbial cross-feeding systems.

### A Visual Overview of the Cross-Feeding and Chemical Interaction Between PGPR Strains, as Investigated Through Metabolomics in this Study

**Fig. 7:**
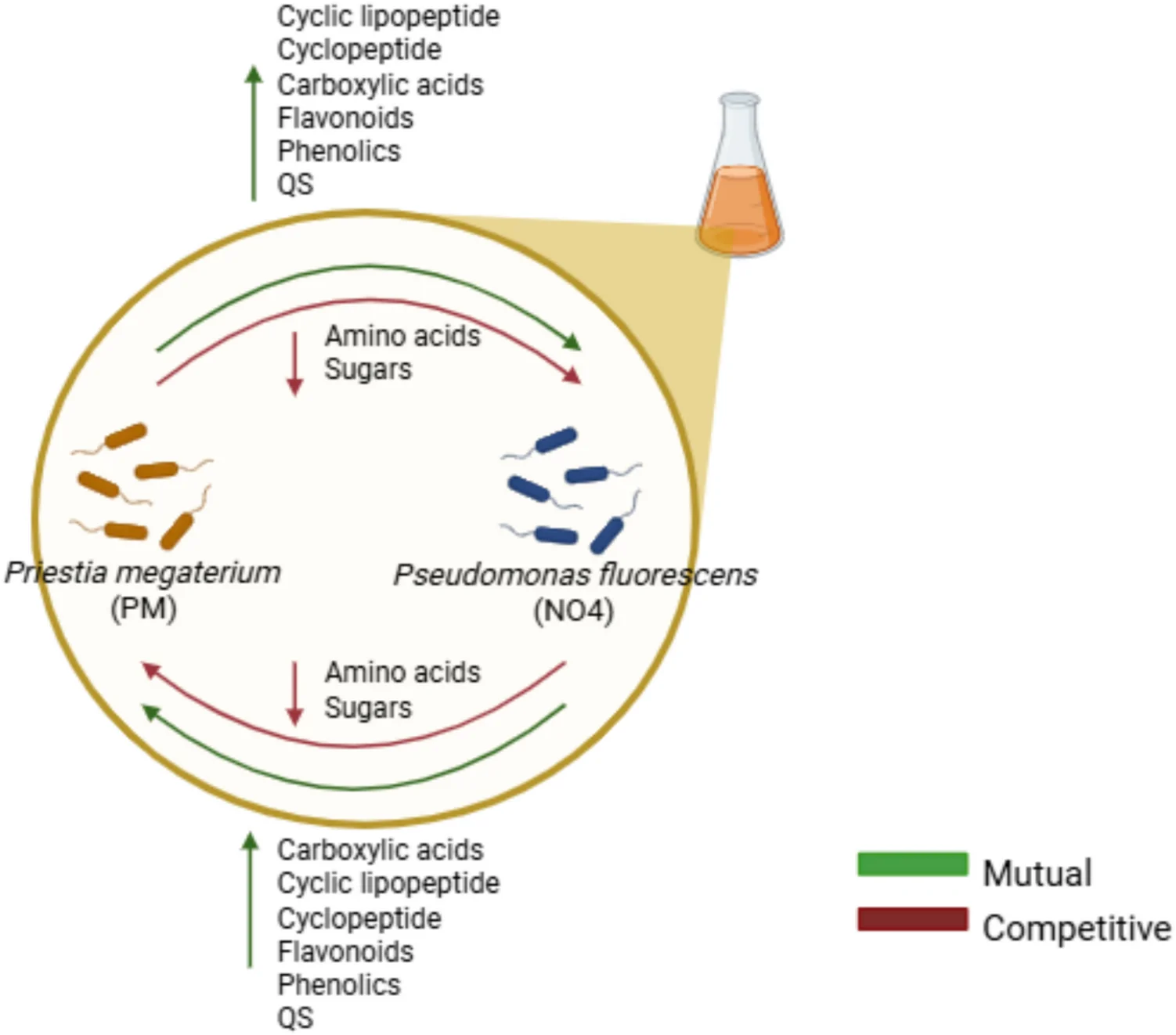
The analysis revealed both mutualistic (indicated by green arrows) and competitive interactions (indicated by red arrows) between the strains. Specifically, the increase in metabolites such as cyclic lipopeptides, flavonoids, phenolics, quorum sensing (QS) compounds molecules, and cyclopeptides were associated with mutualistic interactions, suggesting that these compounds foster beneficial relationships between the PGPR strains. Conversely, a decrease in amino acids and sugars indicated nutrient depletion in the donor media, leading to competitive interactions as the strains competed for limited resources

The results in Fig. 7 illustrate the complex interactions between *P. megaterium* PM and *P. fluorescens* NO4 during cross-feeding. The analysis revealed a dynamic interplay of mutualistic and competitive interactions, as indicated by green and red arrows. A key observation was the inverse relationship between primary and secondary metabolite levels: as amino acids and sugars (primary metabolites) declined due to shared consumption, secondary metabolites such as cyclic lipopeptides, flavonoids, phenolics, quorum sensing (QS) compounds, and cyclopeptides increased. This shift suggests a metabolic adaptation in response to nutrient depletion. Competitive interactions arose primarily from the depletion of essential nutrients in the donor media, particularly sugars and amino acids critical for microbial growth. Despite this, mutualistic interactions were evident through the exchange and accumulation of secondary metabolites. These compounds are known to facilitate biofilm formation, microbial communication, stress tolerance, and cooperative behaviour promoting beneficial relationships between the PGPR strains.

Figure 7 visually reinforces these findings by highlighting the directional exchange of metabolites and the resulting interaction types. The observed balance between nutrient competition and metabolite-mediated cooperation underscores the ecological significance of cross-feeding in shaping PGPR behaviour. Ultimately, such interactions contribute to microbial stability, resilience, and enhanced plant growth promotion in the rhizosphere.

## Discussion

Microbial interactions are central to shaping rhizosphere communities, influencing growth, nutrient cycling, and metabolic activity. Depending on nutrient availability and environmental conditions, these interactions may be cooperative or competitive. This study reveals a complex interplay between *P. megaterium* PM and *P. fluorescens* NO4 during cross-feeding, marked by both mutualism and competition. An inverse relationship was observed between primary and secondary metabolites: the depletion of amino acids and sugars coincided with an increase in secondary metabolites such as cyclic lipopeptides, flavonoids, phenolics, quorum-sensing molecules, and cyclopeptides suggesting metabolic adaptation to nutrient stress. Recent research underscores the importance of nutrient availability and chemical signalling in modulating microbial interactions and shaping community structure and function [19, 42]. In this context, competition stemmed from shared nutrient depletion, while mutualism was driven by the exchange of secondary metabolites that support biofilm formation, communication, stress tolerance, and cooperative behaviour. This balance between competition and cooperation underscores the ecological significance of cross-feeding in modulating PGPR behaviour, enhancing microbial stability, resilience, and plant growth promotion in the rhizosphere.

### Nutrient Competition and Growth Inhibition in Cross-Feeding Interactions

The observed decrease in the microbial growth of the cross-fed receiver species suggests that the interactions among the two PGPR strains were not conducive to enhanced growth under the experimental conditions (Fig. 2A and B). This decrease in growth could be attributed to the lack of nutrient availability, stemming from the experimental setup where the receiver species was introduced into the media already previously occupied by the donor species. This situation likely arises because the donor has consumed some nutrients in the medium. This aligns with previous research indicating that nutrient scarcity often leads to competitive inhibition or resource limitation among microbes [43]. Nutrient competition is particularly pronounced in resource-deficient environments like the rhizosphere, where microbes compete for carbon-rich root exudates that serve as primary energy sources [44].

Nutrient availability is essential for microbial proliferation and metabolic activity; scarcity can result in competitive inhibition or resource limitation among microbes, as mentioned earlier. In nutrient-deficient environments, they may face challenges in accessing the resources required for optimal growth and reproduction, leading to reduced overall performance. For example, Gonzalez and Aranda [45] illustrated that when essential nutrients such as amino acids and sugars are depleted, microbial growth is significantly impaired. This is consistent with the findings of this study, where a reduced growth of the receiver species was observed under the cross-feeding conditions due to limited access to essential resources.

### Cross-Feeding: Metabolite Exchange and Signalling Adaptations in Microbial Interactions

Cross-feeding is a key mechanism facilitating microbial interactions in the rhizosphere. It involves the exchange of metabolites, whereby byproducts or intermediates produced by one species serve as substrates for another. In this study, mannitol accumulation (Figs. 3B and 5A) in cross-fed samples (N04_PM) suggests a targeted metabolic adaptation by *P. fluorescens* NO4, enabling *P. megaterium* PM to use it as a carbon source. This observation aligns with Agudelo et al. [46] research showing that cross-feeding interactions enhance microbial diversity by allowing different species to coexist on limited resources.

Metabolite exchange during cross-feeding can occur through active transport or passive diffusion mechanisms. While bacteria are not inherently “leaky,” metabolite externalisation often occurs via transporters that release excess metabolites to maintain homeostasis [47]. For instance, mannitol externalisation by *P. fluorescens* NO4 in Fig. 5A may represent an adaptive strategy to optimise resource utilisation while fostering cooperative interactions.

The accumulation of pyocyanin (Figs. 4B and 5B) in cross-fed samples (PM_N04) highlights its role as a secondary metabolite involved in microbial signalling. Pyocyanin has been shown to modulate redox balance and facilitate iron acquisition under nutrient-limited conditions [48]. These findings suggest that cross-feeding interactions are not merely resource-sharing mechanisms but also involve complex signalling networks that regulate microbial fitness and functionality. Furthermore, other metabolites overlap across treatments, suggesting that certain metabolic pathways are universally utilised among different microbial species under varying conditions. This finding aligns with existing literature indicating that cross-feeding interactions can promote microbial diversity by allowing various species to coexist on limited resources [49, 50]. The ability of microbes to share resources through cross-feeding can lead to increased community stability, inducing systemic resistance and resilience against environmental fluctuations.

The emergence of carboxylic acids such as Basiliskamide A, Bacillaene, and Bacilysocin in PM_NO4 (where *P. fluorescens* NO4 is grown in the media of *P. megaterium* BM) is a result of cross-feeding interactions, which facilitate the exchange of metabolites, including carboxylic acids, between microbial species [51]. Carboxylic acids are commonly transferred during cross-feeding, serving as both nutrients and signalling molecules that shape microbial community dynamics [52, 53]. Such metabolite exchange is a well-documented aspect of bacterial interactions and is critical for establishing metabolic niches and promoting community stability [54, 55]. However, the specific production of specialised carboxylic acids like Basiliskamide A, Bacillaene, and Bacilysocin is typically associated with *Bacillus* and related genera, suggesting that their presence in PM_NO4 likely results from metabolic adaptations or residual biosynthetic activity influenced by the donor media (PM) environment.

Signalling molecules such as SA also play critical roles in these interactions. The emergence of SA in cross-fed samples highlights its role as a key signalling molecule involved in microbial interactions. SA plays a crucial role in plant–microbe communication by inducing systemic resistance against pathogens and modulating defense responses under environmental stresses [56, 57]. Its presence suggests activation of signalling pathways that may enhance microbial communication within the rhizosphere. Additionally, SA can act as a precursor for other metabolic processes linked to stress tolerance and immune responses [55–58]. The ability of microbes to modulate their metabolite profiles based on environmental pressures reflects their ecological adaptability. These findings further highlight the dual role of metabolites as both nutrients and signalling molecules within microbial communities.

### Metabolic Pathways Altered by Cross-Feeding

The metabolic differences observed between cross-fed and control samples emphasise the significant impact of PGPR interactions on metabolic pathways. Amino acids and sugars, being critical nutrients for energy production and biosynthesis, were depleted in cross-fed samples due to competition between donor and receiver species (Fig. 6A and B). This finding aligns with the Amaya-Gómez et al. [58] report, indicating that PGPR strains often compete for essential resources such as carbon and nitrogen. Interestingly, the emergence of new metabolites such as surfactins (cyclic lipopeptides) and Basiliskamide (carboxylic acids) in cross-fed samples suggests significant metabolic perturbations resulting from PGPR interactions. Surfactins are known for their antimicrobial properties and roles in biofilm formation [59, 60], indicating potential synergistic effects between *P. fluorescens* NO4 and *P. megaterium* PM. Previous studies have demonstrated that optimised nutrient supply can stimulate surfactin production [61, 62], further highlighting its relevance for biotechnological applications.

### Signalling Mechanisms Behind the Shift in Metabolite Production and Its Agricultural Importance

As summarised in Fig. 7, the results reveal a dynamic interplay of mutualistic and competitive interactions during cross-feeding between *P. megaterium* PM and *P. fluorescens* NO4. The inverse relationship between declining primary metabolites (amino acids and sugars) and increased secondary metabolites (cyclic lipopeptides, flavonoids, phenolics, quorum-sensing compounds, carboxylic acids, and cyclopeptides) indicates metabolic adaptation to nutrient depletion in the donor medium. Primary metabolites such as amino acids and sugars are essential for bacterial growth as they serve as building blocks [63, 64]. Amino acids are essential for protein synthesis [65], while sugars provide energy through glycolysis and other metabolic pathways [66]. However, under nutrient-limited conditions, bacteria tend to produce secondary metabolites [67], which were observed in the study. This transition is induced by complex regulatory systems that microbes use to sense environmental cues, such as nutrient depletion or competitive pressures from neighbouring microbes. For example, quorum-sensing molecules act as signalling compounds that regulate gene expression in response to population density, triggering the biosynthesis of secondary metabolites. These signalling pathways often involve transcriptional regulators that modulate the expression of genes involved in secondary metabolite biosynthesis [67, 68].

The observed increase in secondary metabolites alongside a decrease in primary metabolites in cross-fed samples suggests that these compounds are central to the bacteria’s adaptive strategies under nutrient-limited conditions. Secondary metabolites often confer ecological advantages by mediating interactions with other microorganisms. Their elevated production supports sustainable pest management strategies through biocontrol applications. For instance, cyclic lipopeptides and phenolics exhibit antimicrobial properties that help suppress competing microbes in the environment [69, 70]. Liu et al. [71] reported that phenolics such as phenylalanine derivatives produced by *P. megaterium* P-NA14 (from potato) strongly inhibited *Staphylococcus aureus*, while *P. megaterium* D-HT207 (from dendrobium) targeted *Escherichia coli*. Similarly, Nielsen et al. [72] showed that cyclic lipopeptides produced by P*. fluorescens* strains exhibited potent biosurfactant and antibiotic activities against root-pathogenic fungi. Additionally, cyclopeptides and flavonoids may function as signalling molecules or protectants under oxidative stress. The production of these secondary metabolites is often tightly regulated by nutrient availability [73, 74]. Therefore, the shift from primary to secondary metabolism under nutrient stress has important implications for sustainable agriculture, including optimised resource use, reduced reliance on pesticides and fertilisers, and improved ecological resilience.

## Conclusion

This study highlights the intricate metabolic dynamics controlling microbial interactions within the rhizosphere, especially the role of cross-feeding in shaping community ecology. The findings demonstrated that under nutrient-limited conditions, a shift from primary to secondary metabolite production was observed, reflecting the adaptive strategies employed by bacteria in response to environmental signals. The depletion of amino acids and sugars indicated competitive interactions for resources, while cyclic lipopeptides, cyclopeptides, flavonoids, phenolics, and quorum sensing (QS) molecules suggested mutualistic or cooperative interaction between the two species. Secondary metabolites mediate ecological competition and cooperation, underscoring their relevance for developing sustainable microbial consortia. Furthermore, the identification of key metabolites, such as surfactins, salicylic acid, and carboxylic acids, highlights their significance in microbial communication, nutrient acquisition, and plant–microbe interactions. These findings suggest that manipulating nutrient availability can optimise the production of beneficial secondary metabolites and promote synergistic relationships between PGPR strains, potentially reducing the reliance on synthetic pesticides and fertilisers, ultimately enhancing the resilience of agricultural systems. Therefore, by harnessing a deeper understanding of metabolite signalling and nutrient dynamics, future strategies can focus on designing more efficient microbial consortia for sustainable agricultural practices, paving the way for innovative and environmentally conscious approaches to crop production.

## Supplementary Information

Below is the link to the electronic supplementary material.

ESM 1 (DOCX 1.17 MB)

## Data Availability

No datasets were generated or analysed during the current study.
