# Supplementary material for: Metabolomic Insights into Cross-Feeding Interactions Between Priestia megaterium PM and Pseudomonas fluorescens NO4: Unveiling Microbial Communication in Plant Growth-Promoting Rhizobacteria
Source: Microb Ecol. 2025 Jul 17;88(1):76. doi: 10.1007/s00248-025-02577-2 (PMC12270958; doi:10.1007/s00248-025-02577-2)
Supplement: Supplementary file 1 — (DOCX 1.17 MB) [file 248_2025_2577_MOESM1_ESM.docx]

**Supplementary tables**

**Metabolomic insights into cross-feeding interactions between** ***Priestia megaterium* PM and *Pseudomonas fluorescens* NO4: Unveiling microbial communication in plant growth-promoting rhizobacteria**

**Nompumelelo R. Sibanyoni^1,2^, Lizelle A. Piater^2^, Pavel Kerchev^4^, Ntakadzeni E. Madala^5^ and Msizi I. Mhlongo^1,2*^**

^1^Imbewu Metabolomics Research Group, Department of Biochemistry, Faculty of Science, University of Johannesburg, Auckland Park, 2006, South Africa.

^2^Research Centre for Plant Metabolomics, Faculty of Science, University of Johannesburg, Auckland Park, 2006, South Africa

^3^Ubuntu Lab, Department of Biochemistry, Faculty of Science, University of Johannesburg, Auckland Park, 2006, South Africa.

^4^Department of Stress, Development and Signalling in Plants, Estación Experimental del Zaidín, CSIC, Profesor Albareda 1, 18008, Granada, Spain.

^5^Department of Biochemistry and Microbiology, Faculty of Science, Engineering and Agriculture, University of Venda, Thohoyandou, South Africa
*Correspondence: E-mail: [mmhlongo@uj.ac.za](mailto:mmhlongo@uj.ac.za); Tel.: +27-11-559-4573

**Supplementary Table 1.** The optical density measurements for PM: PM served as the control, and NO4_PM was cross-fed with metabolites produced by NO4. These measurements were taken at six-hour time intervals (0-36 hr), allowing for a detailed comparison of PM growth dynamics under each condition.

| **SAMPLE** | **TIME** | **REP 1** | **REP 2** | **REP 3** | **AVERAGE** |
| --- | --- | --- | --- | --- | --- |
| PM | 0 | 0,1 | 0,1 | 0,1 | 0,1 |
| PM | 6 | 0,864 | 0,884 | 0,793 | 0,847 |
| PM | 12 | 1,176 | 1,249 | 1,224 | 1,216333 |
| PM | 18 | 1,289 | 1,296 | 1,309 | 1,298 |
| PM | 24 | 1,227 | 1,237 | 1,21 | 1,224667 |
| PM | 30 | 1,208 | 1,287 | 1,296 | 1,263667 |
| PM | 36 | 1,936 | 1,85 | 1,814 | 1,866667 |
| NO4_PM | 0 | 0,1 | 0,1 | 0,1 | 0,1 |
| NO4_PM | 6 | 0,492 | 0,463 | 0,505 | 0,486667 |
| NO4_PM | 12 | 0,818 | 0,809 | 0,743 | 0,79 |
| NO4_PM | 18 | 1,047 | 0,967 | 1,042 | 1,018667 |
| NO4_PM | 24 | 1,125 | 1,151 | 1,186 | 1,154 |
| NO4_PM | 30 | 1,121 | 1,102 | 1,099 | 1,107333 |
| NO4_PM | 36 | 1,347 | 1,329 | 1,181 | 1,285667 |

**Supplementary Table 2.** The optical density measurements for NO4: NO4 served as the control, and PM_NO was cross-fed with metabolites produced by PM. These measurements were taken at six-hour time intervals (0-36 hr), allowing for a detailed comparison of PM growth dynamics under each condition.

| **SAMPLE** | **TIME** | **REP 1** | **REP 2** | **REP 3** | **AVERAGE** |
| --- | --- | --- | --- | --- | --- |
| NO4 | 0 | 0,1 | 0,1 | 0,1 | 0,1 |
| NO4 | 6 | 0,619 | 0,6466 | 0,673 | 0,6462 |
| NO4 | 12 | 1,125 | 1,119 | 1,148 | 1,130667 |
| NO4 | 18 | 1,5222 | 1,4677 | 1,506 | 1,498633 |
| NO4 | 24 | 1,606 | 1,621 | 1,604 | 1,610333 |
| NO4 | 30 | 1,752 | 1,732 | 1,729 | 1,737667 |
| NO4 | 36 | 1,744 | 1,773 | 1,815 | 1,777333 |
| PM_NO4 | 0 | 0,1 | 0,1 | 0,1 | 0,1 |
| PM_NO4 | 6 | 0,637 | 0,66 | 0,6632 | 0,6534 |
| PM_NO4 | 12 | 0,854 | 0,941 | 0,952 | 0,915667 |
| PM_NO4 | 18 | 1,141 | 1,175 | 1,061 | 1,125667 |
| PM_NO4 | 24 | 1,254 | 1,244 | 1,142 | 1,213333 |
| PM_NO4 | 30 | 1,334 | 1,343 | 1,208 | 1,295 |
| PM_NO4 | 36 | 1,419 | 1,339 | 1,341 | 1,366333 |

**Supplementary Table 3.** List of the significantly discriminant metabolites (VIP>0.7 and *p*<0.05) 74 and their chromatographic & spectral characteristics based on the LC-MS analysis obtained for 75 MCF-1 treated (Ad → Br) and control sets of *Priestia*.

| **No** | **Compound name** | **Chemical formula** | **Rt** | **m/z** | **Adduct** | **Fragments (m/z)** | **BM** | **NO4** | **BM_NO4** | **NO4_BM** |
| --- | --- | --- | --- | --- | --- | --- | --- | --- | --- | --- |
| 1. | Surfactin A | C51H89N7O13 | 13.15 | 1008.60291 | [M+H]+ | 227.1319, 685.3660 | ✔ |  | ✔ | ✔ |
| 2. | Surfactin B-C13 | C50H87N7O13 | 13.59 | 994.6412 | [M+H]+ | 667.4604, 685.3730 | ✔ |  | ✔ | ✔ |
| 3. | Surfactin B-C15 | C52H91N7O13 | 13.32 | 1020.64820 | [M+H]+ |  | ✔ |  | ✔ | ✔ |
| 4. | Surfactin C | C53H93N7O13 | 13.82 | 1036.6899 | [M+H]+ | 685.30 | ✔ |  | ✔ |  |
| 5. | Surfactin D | C54H95N7O13 | 13.61 | 1050.57959 | [M+H]+ | 199.11, 227.11 | ✔ |  |  |  |
| 6. | Iturin A8 | C51H80N12O14 | 13.41 | 1107.6499 | [M+Na]+ | 562.2994 | ✔ |  |  |  |
| 7. | 3-Hydroxyflavone | C15H10O3 | 11.94 | 237.07175 | [M+H]+ | 181.0792 | ✔ |  | ✔ | ✔ |
| 8. | Pyroglutamylproline | C10H14N2O4 | 11.94 | 227.085 | [M+H]+ |  | ✔ | ✔ |  | ✔ |
| 9. | Methyl Orsellinate | C9H10O4 | 15.32 | 181.06 | [M+H]+ | 150.03 | ✔ |  |  |  |
| 10. | D-Mannitol | C6H14O6 | 11.94 | 227.09 | [M+K]+ | 116.03 | ✔ | ✔ |  |  |
| 11. | L-methionine sulfone | C5H11NO4S | 6.87 | 182.08530 | [M+H]+ | 182.14 | ✔ | ✔ |  |  |
| 12. | 7-O-Succinyl macrolactin A | C28H38O8 | 1.01 | 524.14230 | [M+Na]+ | 385.16 | ✔ |  |  |  |
| 13. | 7-O-succinyl macrolactin F | C28H38O8 | 8.63 | 488.24960 | [M+H]+ | 407.20 | ✔ |  |  |  |
| 14. | Bacillaene | C34H48N2O6 | 5.79 | 583.30050 | [M+H]+ | 223.66, 432.21 | ✔ |  |  | ✔ |
| 15. | Bacilysocin | C21H43O9P | 8.46 | 471.36590 | [M+H]+ | 300.21, 331.12 | ✔ |  |  |  |
| 16. | Basiliskamide A | C23H31NO4 | 7.99 | 385.26470 | [M+H]+ | 217.12 | ✔ |  |  |  |
| 17. | cyclo(L-Leu-L-4-hydroxy-Pro) | C11H18N2O2 | 10.37 | 227.17900 | [M+H]+ | 228.20 | ✔ |  |  |  |
| 18. | Swietenocoumarin F | C17H18O6 | 1.18 | 319.15470 | [M+Na]+ | 271.10 | ✔ | ✔ | ✔ |  |
| 19. | N-Methyltryptamine | C11H14N2 | 5.21 | 175.12230 | [M+H]+ | 118.07, 144.06, 158.10, 175.15 | ✔ | ✔ |  |  |
| 20. | Adenosine Phosphate | C10H14N5O7P | 3.49 | 348.08 | [M+H]+ | 137.07 | ✔ | ✔ |  |  |
| 21. | Chorismic acid | C10H10O6 | 13.23 | 227.10 | [M+H]+ | 173.02, 192, 209.04 | ✔ |  |  |  |
| 22. | Pentosalen | C16H14O4 | 3.51 | 271.13 | [M+H]+ | 203.02 | ✔ |  |  |  |
| 23. | Cyclo(proline-leucine) | C11H18N2O2 | 9.85 | 211.14700 | [M+H]+ | 160. 197 | ✔ |  |  |  |
| 24. | Cyclo-prolylglycine | C7H10N2O2 | 9.66 | 155.08180 | [M+H]+ |  | ✔ |  |  |  |
| 25. | Tryptophan | C11H12N2O2 | 5.09 | 205.09650 | [M+H]+ | 118.09, 144.10 | ✔ |  |  |  |
| 26. | Macrolactin A | C24H34O5 | 1.07 | 402.81480 | [M+H]+ | 332,10, 344,15 | ✔ |  |  |  |
| 27. | Phenazine | C12H8N2 | 11.94 | 181.08440 | [M+H]+ | 180.07 | ✔ | ✔ | ✔ |  |
| 28. | Pseudobactin | C42H60N12O16 | 8.62 | 325.12220 | [M+H]+ | 204.13, 230.15 |  | ✔ |  |  |
| 29. | Salicylic Acid | C7H6O3 | 9.64 | 139.08850 | [M+H]+ | 111.05 | ✔ | ✔ |  |  |
| 30. | Rhamnose | C6H12O5 | 6.17 | 187.07170 | [M+Na]+ | 169.000 | ✔ |  |  |  |
| 31. | Ascorbic acid | C6H8O6 | 13.144 | 177.0672 | [M+H]+ | 158.0 | ✔ |  |  |  |
| 32. | Pregabalin | C8H17NO2 | 6.42 | 160.10580 | [M+H]+ | 142.09, 143.09 |  | ✔ |  |  |
| 33. | Liquiritigenin | C15H12O4 | 0.,94 | 257.11500 | [M+H]+ |  | ✔ |  |  |  |
| 34. | 3-Nitro-L-tyrosine | C9H10N2O5 | 11.94 | 227.08480 | [M+H]+ | 168.08, 210.07 |  | ✔ | ✔ |  |
| 35. | Coronatine | C18H25NO4 | 14.24 | 339.18660 | [M + Na]+ |  |  | ✔ |  |  |
| 36. | Mangotoxin |  | 6.17 | 600.30190 | [M + Na]+ |  |  | ✔ |  |  |
| 37. | Syringomycin | C53H85ClN14O17 | 9.64 | 139.08850 | [M + K]+ |  |  | ✔ |  |  |
| 38. | 2,4-Diacetylphloroglucinol | C10H10O5 | 1.17 | 235.02240 | [M + Na]+ | 125.07 |  | ✔ |  |  |
| 39. | Fenofibrate | C20H21ClO4 | 1.33 | 361.17 | [M+H]+ | 233.37, 235.08 |  | ✔ |  |  |
| 40. | (2R)-5-methoxy-2-methyl-2,3,8,9-tetrahydrofuro[2,3-h]chromen-4-one | C13H14O4 | 1.00 | 233.07 | [M+Na]+ | 190.13 |  |  | ✔ |  |
| 41. | D-Fructose | C6H12O6 | 0.93 | 203.05890 | [M+Na]+ | 200.91 | ✔ |  | ✔ |  |
| 42. | Sucrose | C12H22O11 | 1.01 | 360.1451 | [M+NH4]+ | 146.06 | ✔ |  | ✔ | ✔ |

**Supplementary figures**


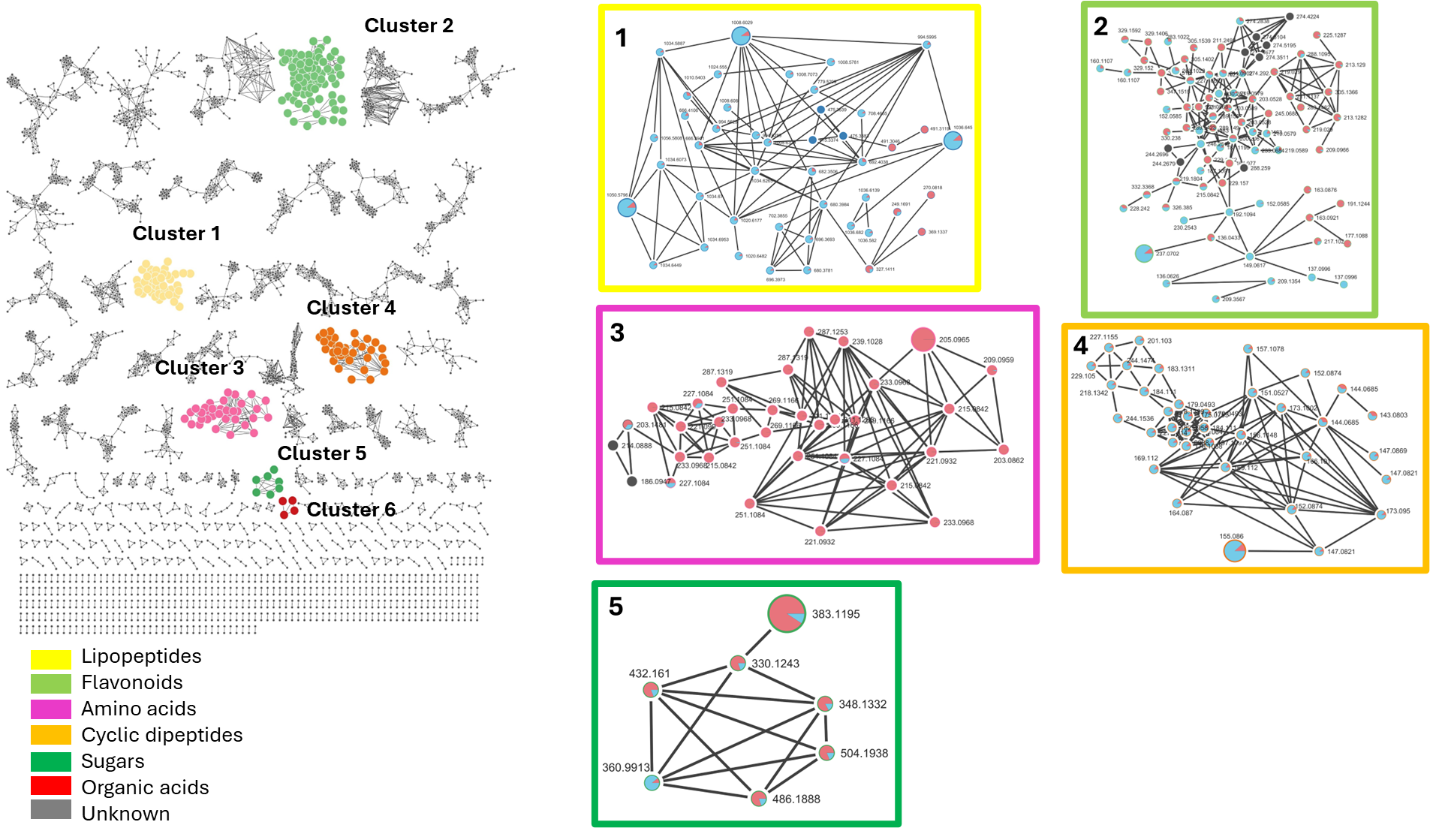


**Figure S1: Molecular networking of positive electrospray ionization (ESI+) spectra obtained for the PGPR-PGPR (*P. megaterium* PM and *P. fluorescens* NO4) cross-feeding interactions.** The zoom-in snaps show the different metabolite classes present in the interactions.

**
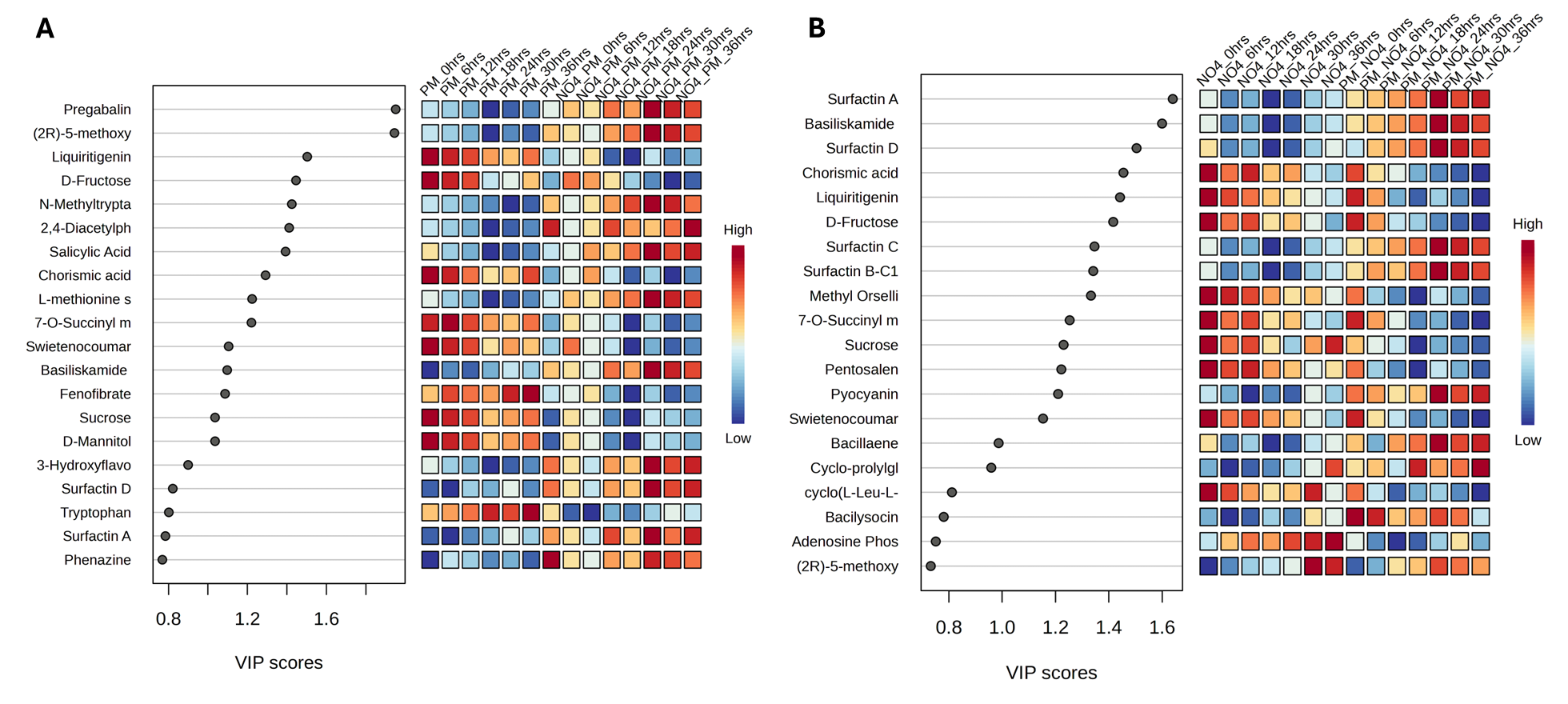
**

**Figure S2: VIP score-plots obtained from the PLS-DA analysis. The VIP scores display discriminant features for the effect of PGPR on another PGPR (*P. megaterium* PM and P*. fluorescens* NO4). A.** *P. megaterium* PM is the receiver species, and *P. fluorescens* NO4 is the donor species. **B**. *P. fluorescens* NO4 is the receiver species, and *P. megaterium* PM is the donor species. Selected metabolites (VIP score ≥ 0.5) in cross-fed samples were compared to their control. The figures show differential up-/down-regulation of significant metabolites in cross-fed compared to control samples. The data displayed above were generated in MetaboAnalyst and were median-normalised, log-transformed, and Pareto-scaled.
